# Understanding Nonadherence to Tuberculosis Medications in India Using Urine Drug Metabolite Testing: A Cohort Study

**DOI:** 10.1093/ofid/ofab190

**Published:** 2021-05-05

**Authors:** Ramnath Subbaraman, Beena E Thomas, J Vignesh Kumar, Kannan Thiruvengadam, Amit Khandewale, S Kokila, Maya Lubeck-Schricker, M Ranjith Kumar, Gunjan Rahul Gaurkhede, Apurva Shashikant Walgude, J Hephzibah Mercy, Jagannath Dattatraya Kumbhar, Misha Eliasziw, Kenneth H Mayer, Jessica E Haberer

**Affiliations:** 1 Department of Public Health and Community Medicine, Tufts University School of Medicine, Boston, Massachusetts, USA; 2 Division of Geographic Medicine and Infectious Diseases, Tufts Medical Center, Boston, Massachusetts, USA; 3 Department of Social and Behavioural Research, ICMR-National Institute for Research in Tuberculosis, Chennai, India; 4 The Fenway Institute, Fenway Health and Department of Medicine, Beth Israel Deaconess Medical Center and Harvard Medical School, Boston, Massachusetts, USA; 5 Center for Global Health, Massachusetts General Hospital and Department of Medicine, Harvard Medical School, Boston, Massachusetts, USA

**Keywords:** adherence, alcohol use, HIV, India, tuberculosis

## Abstract

**Background:**

Poor adherence to tuberculosis (TB) treatment is associated with disease recurrence and death. Little research has been conducted in India to understand TB medication nonadherence.

**Methods:**

We enrolled adult drug-susceptible TB patients, approximately half of whom were people with human immunodeficiency virus (PWH), in Chennai, Vellore, and Mumbai. We conducted a single unannounced home visit to administer a survey assessing reasons for nonadherence and collect a urine sample that was tested for isoniazid content. We described patient-reported reasons for nonadherence and identified factors associated with nonadherence (ie, negative urine test) using multivariable logistic regression. We also assessed the association between nonadherence and treatment outcomes.

**Results:**

Of 650 participants in the cohort, 77 (11.8%) had a negative urine test. Nonadherence was independently associated with daily wage labor (adjusted odds ratio [aOR], 2.7; confidence interval [CI], 1.1–6.5; *P* = .03), the late continuation treatment phase (aOR, 2.0; CI, 1.1–3.9; *P* = .03), smear-positive pulmonary disease (aOR, 2.1; CI, 1.1–3.9; *P* = .03), alcohol use (aOR, 2.5; CI, 1.2–5.2; *P* = .01), and spending ≥30 minutes collecting medication refills (aOR, 6.6; CI, 1.5–29.5; *P* = .01). People with HIV reported greater barriers to collecting medications than non-PWH. Among 167 patients reporting missing doses, reported reasons included traveling from home, forgetting, feeling depressed, and running out of pills. The odds of unfavorable treatment outcomes were 4.0 (CI, 2.1–7.6) times higher among patients with nonadherence (*P* < .0001).

**Conclusion:**

Addressing structural and psychosocial barriers will be critical to improve TB treatment adherence in India. Urine isoniazid testing may help identify nonadherent patients to facilitate early intervention during treatment.

## Teaser

We evaluated adherence to tuberculosis medications in 650 Indian patients by conducting urine isoniazid tests collected during unannounced home visits. Structural barriers to collecting medication refills and psychosocial barriers emerged as the most important factors contributing to medication nonadherence.

Poor adherence to active tuberculosis (TB) treatment is associated with increased disease recurrence and death [1–3]. A meta-analysis of recent clinical trials suggests that missing more than 10% of doses during drug-susceptible TB treatment is associated with approximately 6-fold increased risk of unfavorable outcomes, including disease recurrence [2]. Studies in programmatic conditions similarly reveal higher disease recurrence for TB patients with poor adherence [3]. A modeling study suggests that reducing nonadherence could have larger epidemiological impact on TB incidence than decreasing loss to follow-up during treatment in high-burden countries [4].

Despite its importance, there has been little research that has evaluated factors contributing to TB medication nonadherence in high-burden settings such as India, which has the world’s largest epidemic [5]. In the few previous studies in India, researchers have identified clinical (eg, medication adverse effects [6–8], symptom improvement [6]), psychosocial (eg, alcohol use [6, 7, 9], stigma [6, 7]), structural (eg, distance from clinic [6–10], migration [6], work-related challenges [6, 8, 10]), and health system (eg, noncooperative staff [6], drug stockouts [9]) barriers that contribute to nonadherence.

These previous studies have methodological limitations including small samples [6–8]. Some studies reportedly assessing adherence actually measured loss to follow up from treatment, rather than missed doses, which is a behavior with potentially different contributing factors and clinical implications [11–14]. None of these studies used rigorous approaches for measuring adherence, such as monitoring with digital adherence technologies (DATs) [15] or drug metabolite testing [16, 17]—strategies that have been used to understand adherence in human immunodeficiency virus (HIV) and other conditions [18].

Moreover, prior studies were conducted in the context of directly observed therapy (DOT), usually using a facility-based approach, in which patients visited clinics where healthcare providers observed dose ingestion. In recent years, concern has been growing regarding the patient and health system burden, ethical limitations, and effectiveness of DOT [15, 19–22]. At the same time, in 2014, India’s National TB Elimination Program (NTEP) began introducing daily medication dosing for all drug-susceptible TB patients, in place of a prior thrice-weekly dosing regimen [23]. This shift led to concerns about the increased burden of having patients visit clinics daily, rather than thrice weekly [23]. As a result, the NTEP has transitioned away from DOT and towards use of self-administered therapy ([SAT] ie, patients taking pills themselves) or monitoring with 99DOTS, a cellphone-based DAT [23]. No recent studies have evaluated causes of TB treatment nonadherence in India with SAT or monitoring with DATs.

In this manuscript, we investigate TB medication nonadherence using data from a cohort study conducted in 3 Indian cities among people with HIV (PWH) and non-PWH. Adherence was assessed by urine testing for isoniazid during unannounced home visits. Although the study’s primary goal, for which results have been reported [16], was to understand 99DOTS’ accuracy for measuring adherence, the data also provide insights into causes of nonadherence. In this study, our aims are as follows: to identify clinical, structural, and psychosocial factors associated with nonadherence; describe patient-reported reasons for missed doses collected using a structured questionnaire; and assess the association between nonadherence and TB treatment outcomes.

## METHODS

### Ethics Approvals

The study protocol was approved by ethics committees at the National Institute for Research in TB (Chennai, India), Brigham and Women’s Hospital (Boston, MA), and Tufts University (Boston, MA).

### Patient Consent Statement

We obtained written informed consent from all participants enrolled in this study. 

### Study Setting and Care Delivery Approaches

To enroll a geographically diverse cohort, we recruited TB patients from 3 cities with a relatively high TB burden: Mumbai, Chennai, and Vellore [24, 25]. Almost all patients in Mumbai were HIV-negative and recruited from 11 DOT centers with some of the highest patient volumes. Patients in Chennai and Vellore were PWH recruited from the 5 largest HIV antiretroviral therapy (ART) centers in these cities. As such, in our analyses, HIV status not only serves as a marker of this comorbidity, but it also serves as a proxy for differences in geographic sites and care delivery approaches, as described below.

Tuberculosis care delivery approaches varied between DOT and ART centers. Before 2014, PWH collected TB medications at DOT centers near their homes, similar to non-PWH. With subsequent introduction of daily TB medication dosing using a SAT- or DAT-monitored approach, PWH started to collect TB medication refills at ART centers concurrently with ART refills (in a “single window”), usually on a monthly basis [26]. Although DOT centers are decentralized in primary health centers in India, there are fewer ART centers that are usually located in tertiary hospitals. As such, the single window approach increased convenience by consolidating HIV and TB pharmacy services; however, it usually increased the distances PWH needed to travel to collect TB medications.

### Patient Recruitment and Data Collection

From August 2017 to February 2019, we sequentially recruited adult patients (18 years of age and older) from the selected clinics who were taking drug-susceptible TB therapy monitored with 99DOTS. To understand variability in adherence during treatment, we initially enrolled patients visiting clinics not only when they started treatment but also when they collected monthly medication refills, excluding the last treatment month. After exhausting the pool of patients already taking treatment, we continued to enroll those initiating treatment but randomly assigned participants to receive the home visit in the intensive (first 2 months) or the continuation (last 4 months) phase to ensure distribution of home visits across phases.

Informed consent was obtained upon enrollment. Based on a socioecological framework [27], a baseline questionnaire was designed to collect data on demographic, socioeconomic, structural, and psychosocial factors—including the brief alcohol use disorder identification test (AUDIT-C) [28]. Patients became eligible for a home visit 3 weeks after enrollment or after reaching the continuation phase, for those randomized to receive a home visit in that phase. A random number generator was used to select the exact day of the visit.

We tried to minimize changes in adherence behavior in response to study interactions (Hawthorne effect). We conducted a single home visit with every patient under the assumption that patients may modify their behavior with repeated visits. Although this single visit limits understanding of adherence longitudinally for each patient, home visits for the cohort were distributed throughout the treatment course, providing reasonable representation of adherence for the population. Waiting at least 3 weeks for the home visit likely reduced the impact of short-term behavioral changes related to the initial study enrollment interaction. The home visit was also conducted without prior notice to minimize the likelihood of patients taking medications solely in anticipation of the visit. During the visit, we asked patients questions modified from the AIDS Clinical Trials Group adherence questionnaire to assess reasons for nonadherence and collected a urine sample that was tested using the IsoScreen assay [29].

### Interpretation of the Urine Test Result

IsoScreen is a validated assay based on the Arkansas (Potts-Cozart) method. Assay reagents change color through reactions with isoniazid or its metabolite acetylisoniazid [30–32]. Yellow, green, and blue/purple test results suggest no detection, low-level detection, and high-level detection, respectively [30, 31]. These 3 color categories can be used to construct 2 different binary adherence outcomes, which we will refer to as “nonadherence” and “suboptimal adherence.”

When interpreting IsoScreen results by comparing any color change (green or blue/purple) versus no color change (yellow), validation studies show that approximately 100%, 83%, and 11% of patients will have positive test results 24, 48, and 72 hours, respectively, after last ingestion of isoniazid [30, 31]. We defined nonadherence as comprising yellow test results (in comparison to green or blue/purple results), indicating high likelihood of not having taken doses for 48 to 72 hours or longer. This approach has <11% chance of misclassifying patients who did not take a dose within the last 72 hours as being adherent.

Previous validation studies also showed that, whereas blue/purple color indicates medication ingestion within the last 24 hours, green indicates the last dose ingestion occurred 24 to 48 hours earlier, suggesting a dose was missed [30, 31]. Therefore, we defined suboptimal adherence as comprising yellow or green test results (in comparison to blue/purple results), indicating high likelihood of not having taken a dose for 24 hours or longer. By including green color as comprising a negative result, we increased the test’s sensitivity for detecting poor adherence, although this approach may misclassify a small proportion of patients (<17%) who are correctly taking their medications as being suboptimally adherent [31]. Given the strengths and deficiencies of each approach to classifying urine test results, and the fact that each approach provides slightly different insights into adherence behavior, we conducted separate analyses using each adherence outcome.

### Analyses

We used JMP Pro 15 (SAS Institute, Inc., Cary, NC) to perform univariable and multivariable logistic regression analyses to identify clinical, structural, and psychosocial factors associated with nonadherence and, separately, suboptimal adherence. All demographic and clinical factors were retained in the multivariable model by design. Other factors were retained if significant at *P* < .2 in the univariable analyses. Modifications to the model to remediate multicollinearity are described in the Supplementary Appendix. We classified the timing of the home visit based on whether it happened in the intensive phase (≤56 days of therapy), early continuation phase (57 to 112 days of therapy), or late continuation phase (>112 days of therapy). We included alcohol use as a binary variable comparing those with moderate or high use (AUDIT-C score of 1 or greater) to no alcohol use (score of 0). For additional insights into nonadherence, we described the proportion of patients reporting various reasons for missing medication doses in the home visit questionnaire.

Finally, we evaluated whether the urine test result was associated with treatment outcomes. We used separate logistic regression analyses to assess whether nonadherence and suboptimal adherence were associated with outcomes of death, loss to follow up, and “unfavorable treatment outcomes”—the latter being a composite outcome including death, loss to follow up, and treatment failure. We did not separately assess the association between nonadherence and treatment failure, because only 3 patients experienced treatment failure.

We obtained outcomes from patients’ treatment cards through study closure in February 2019 and later from Nikshay (the NTEP’s electronic record) in September 2020 (see Supplementary Appendix for details). We analyzed the association between nonadherence or suboptimal adherence and treatment outcomes separately using each data source.

## RESULTS

### Descriptive Statistics

Of 832 patients meeting eligibility criteria, 84 (10%) did not consent for the study or could not enroll because family members collected their medications. Of 748 patients who enrolled, we could not complete home visits for 98 (13%), despite 3 attempts (Supplementary Appendix, Figure S1). Of 650 patients in the final analysis, 77 (11.8%) were nonadherent and 116 (17.8%) were suboptimally adherent. Among PWH, 51 of 303 (16.8%) were nonadherent compared with 26 of 347 (7.5%) HIV-negative TB patients (*P* = .0002). Among PWH, 73 of 303 (24.1%) were suboptimally adherent compared with 43 of 347 (12.4%) HIV-negative TB patients (*P* < .0001).

More than three quarters of patients had a household monthly income of <15 000 Indian rupees ([INR] approximately <200 US dollars [USD]) (Table 1). Approximately half were housewives, students, or unemployed. Most patients were new (formerly category 1) and had smear-positive pulmonary TB. Few were able to walk or bicycle to clinic; most spent 60 minutes or more collecting medication refills.

**Table 1.** Descriptive Characteristics for the Overall Cohort and Disaggregated by HIV Status

Covariates	Overall Cohort^a^ (N = 650)	People With HIV^a^ (N = 303)	HIV Negative^a^ (N = 347)	*P* Value^b^
Demographic Factors				
Gender				
Female	271 (41.7)	102 (33.7)	169 (48.7)	<.001
Male	379 (58.3)	201 (66.3)	178 (51.3)	
Age				
18–29	226 (34.8)	30 (9.9)	196 (56.5)	<.001
30–44	251 (38.6)	154 (50.8)	97 (28.0)	
≥45	173 (26.6)	119 (39.3)	54 (15.6)	
Monthly Income				
INR <7500	244 (37.5)	174 (57.4)	70 (20.2)	<.001
INR 7500–14 999	263 (40.5)	93 (30.7)	170 (49.0)	
INR ≥15 000	143 (22.0)	36 (11.9)	107 (30.8)	
Occupation				
Self-employed	137 (21.1)	81 (26.7)	56 (16.1)	<.001
Government or private sector employment	130 (20.0)	61 (20.1)	69 (19.9)	
Laborer on daily wages	84 (12.9)	46 (15.2)	38 (11.0)	
Housewife, student, or unemployed	299 (46.0)	115 (38.0)	184 (53.0)	
Clinical Factors				
Phase of Therapy				
Intensive phase	178 (27.4)	102 (33.7)	76 (21.9)	.002
Early continuation phase	249 (38.3)	100 (33.0)	149 (42.9)	
Late continuation phase	223 (34.3)	101 (33.3)	122 (35.2)	
Category of TB				
New	504 (77.5)	242 (79.9)	262 (75.5)	.184
Previously treated	146 (22.5)	61 (20.1)	85 (24.5)	
Type of TB				
Extrapulmonary	202 (31.1)	72 (23.8)	130 (37.5)	<.001
Smear-negative pulmonary	77 (11.9)	48 (15.8)	29 (8.36)	
Smear-positive pulmonary	371 (57.1)	183 (60.4)	188 (54.2)	
Structural Factors				
Transport Mode to Clinic				
Walking or bicycle	234 (36.0)	12 (4.0)	222 (64.0)	<.001
Motorcycle or car	44 (6.8)	30 (9.9)	14 (4.0)	
Autorickshaw or taxi	131 (20.2)	33 (10.9)	98 (28.2)	
Public transportation	241 (37.1)	228 (75.3)	13 (3.8)	
Money Spent to Collect Medication Refills				
INR 0–24	233 (35.9)	21 (6.9)	212 (61.1)	<.001
INR 25–49	111 (17.1)	32 (10.6)	79 (22.8)	
INR-50–75	111 (17.1)	68 (22.4)	43 (12.4)	
INR >75	195 (30.0)	182 (60.1)	13 (3.8)	
Time Spent to Collect Medication Refills				
<30 minutes	110 (16.9)	4 (1.3)	106 (30.6)	<.001^c^
30 to 59 minutes	191 (29.4)	7 (2.3)	184 (53.0)	
60 to 239 minutes	180 (27.7)	123 (40.6)	57 (16.4)	
≥240 minutes	169 (26.0)	169 (55.8)	0 (0.0)	
Psychosocial Factors				
Current Tobacco Use				
No	540 (83.1)	261 (86.1)	279 (80.4)	.002
Smokeless tobacco only	51 (7.9)	12 (4.0)	39 (11.2)	
Cigarette or beedi use	59 (9.1)	30 (9.9)	29 (8.4)	
Probable Alcohol Use				
No alcohol use	591 (90.9)	256 (84.5)	335 (96.5)	<.001
Any alcohol use	59 (9.1)	47 (15.1)	12 (3.5)	

Abbreviations: HIV, human immunodeficiency virus; INR, Indian rupees; TB, tuberculosis.

^a^Represents the number of study participants in a category divided by the overall sample or subsample: eg, there are 271 females out of 650participants in the overall cohort; 102 females out of 303 participants with HIV; and 169 females out of 347 participants who are HIV negative.

^b^χ ^2^ was used to assess differences in characteristics between people with HIV and HIV-negative TB patients.

^c^Fisher’s exact test was used to assess differences for time spent to collect medication refills, because some categories had fewer than 5 observations.

Patient characteristics varied significantly by HIV status. It is notable that PWH were substantially more likely to be older, have lower income, and use alcohol. People with HIV also faced substantially greater structural barriers to collecting medication refills, with regard to transport mode, money spent, and time spent on this activity.

### Factors Associated With Nonadherence and Suboptimal Adherence

In the multivariable model with nonadherence as the outcome, participants who were laborers on daily wages, in the late continuation treatment phase, smear-positive pulmonary TB patients, spending 30 or more minutes collecting medications, or using alcohol were at significantly higher odds of being nonadherent (Table 2). In the multivariable model with suboptimal adherence as the outcome, participants who were laborers on daily wages, spending 30 or more minutes collecting medications, or using alcohol were at significantly higher odds of being suboptimally adherent (Supplementary Appendix, Table S2). Participants in the late continuation phase were also at higher odds of being suboptimally adherent, although this association did not quite achieve statistical significance (*P* = .06).

**Table 2.** Factors Associated With Nonadherence to TB Medications (N = 650)

	Descriptive Statistics	Univariable Findings		Multivariable Findings	
Covariates	Proportion With Nonadherence^a^, n (%)	Odds Ratio (95% Confidence Interval)	*P* Value	Odds Ratio (95% Confidence Interval)	*P* Value
Demographic Factors					
Gender					
Female	28 (10.3)	Ref		Ref	
Male	49 (12.9)	1.3 (0.8–2.1)	.31	1.0 (0.5–1.8)	.92
Age					
18–29	19 (8.4)	Ref		Ref	
30–44	41 (16.3)	2.1 (1.2–3.8)	.01*	1.1 (0.6–2.2)	.71
≥45	17 (9.8)	1.2 (0.6–2.4)	.62	0.6 (0.3–1.2)	.15
Monthly Income					
INR <7500	33 (13.5)	Ref		Ref	
INR 7500–14 999	33 (12.5)	0.9 (0.5–1.5)	.74	1.4 (0.8–2.7)	.24
INR ≥15 000	11 (7.7)	0.5 (0.3–1.1)	.08	0.9 (0.4–2.0)	.74
Occupation					
Self-employed	12 (8.8)	Ref		Ref	
Employed in government or private sector	16 (12.3)	1.5 (0.7–3.2)	.35	1.7 (0.7–3.9)	.23
Laborer on daily wages	16 (19.0)	2.5 (1.1–5.5)	.03*	2.7 (1.1–6.5)	.03*
Housewife, student, or unemployed	33 (11.0)	1.3 (0.6–2.6)	.47	1.6 (0.7–3.6)	.26
Clinical Factors					
Phase of therapy					
Intensive phase	20 (11.2)	Ref		Ref	
Early continuation phase	21 (8.4)	0.7 (0.4–1.4)	.33	1.1 (0.5–2.1)	.87
Late continuation phase	36 (16.1)	1.5 (0.8–2.7)	.16	2.0 (1.1–3.9)	.03*
Category of TB					
New	55 (10.9)	Ref		Ref	
Previously treated	22 (15.1)	1.4 (0.9–2.5)	.17	1.4 (0.8–2.5)	.24
Type of TB					
Extrapulmonary	15 (7.4)	Ref		Ref	
Smear-negative pulmonary	11 (14.3)	2.1 (0.9–4.8)	.08	1.9 (0.8–4.7)	.15
Smear-positive pulmonary	51 (13.7)	2.0 (1.1–3.6)	.03*	2.1 (1.1–3.9)	.03*
People With HIV					
No	26 (7.5)	Ref		Ref	
Yes	51 (16.8)	2.5 (1.5–4.1)	.0003*	1.5 (0.6–3.6)	.43
Structural Factors					
Transport Mode to Clinic					
Walking or bicycle	13 (5.6)	Ref			
Motorcycle or car	5 (11.4)	2.2 (0.7–6.5)	.16		
Autorickshaw or taxi	14 (10.7)	2.0 (0.9–4.5)	.08		
Public transportation	45 (18.7)	3.9 (2.0–7.4)	<.0001*		
Money Spent to Collect Medication Refills					
INR 0–24	17 (7.3)	Ref			
INR 25–49	10 (9.0)	1.3 (0.6–2.8)	.58		
INR 50–75	17 (15.3)	2.3 (1.1–4.7)	.02*		
INR >75	33 (16.9)	2.6 (1.4–4.8)	.003*		
Time Spent to Collect Medication Refills					
<30 minutes	2 (1.8)	Ref		Ref	
30 to 59 minutes	19 (9.9)	6.0 (1.4–26.1)	.02*	6.6 (1.5–29.5)	.01*
≥60 minutes	56 (16.0)	10.3 (2.5–43.0)	.001*	9.0 (1.8–44.2)	.007*
Psychosocial Factors					
Current Tobacco Use					
No	59 (10.9)	Ref			
Smokeless tobacco only	4 (7.8)	0.7 (0.2–2.0)	.50		
Cigarette or beedi use	14 (23.7)	2.5 (1.3–4.9)	.006*		
Probable Alcohol Use					
No alcohol use	61 (10.3)	Ref		Ref	
Any alcohol use	16 (27.1)	3.2 (1.7–6.1)	.0003*	2.5 (1.2–5.2)	.01*

Abbreviations: HIV, human immunodeficiency virus; INR, Indian rupees; Ref, reference group; TB, tuberculosis.

^a^Represents the number of study participants with nonadherence in a given category: eg, 28 of 271 females were nonadherent.

*Indicates a statistically significant finding at the 5% level.

### Patient-Reported Reasons for Nonadherence

Of 650 participants who answered the home visit survey, 167 (25.7%) reported missing at least 1 medication dose during therapy. Of these, more than one fifth reported traveling or being away from home, forgetting to take medications, feeling depressed, or running out of pills as contributing to nonadherence (Table 3). The Supplementary Appendix provides details on why participants ran out of pills and medication adverse effects that impacted adherence.

**Table 3.** Patient-Reported Reasons for Missing Tuberculosis (TB) Medication Doses (N = 167)

	Proportion of Patients Reporting This Problem^b^	Frequency With Which Patients Were Affected by This Problem		
Reason for Missing Doses^a^	n (%)	Rarely n (%)	Sometimes, n (%)	Often, n (%)
Traveling or being away from home	67 (40.1%)	37 (22.2%)	19 (11.4%)	11 (6.6%)
Simply forgot	50 (29.9%)	28 (16.8%)	11 (6.6%)	11 (6.6%)
Felt depressed	39 (23.3%)	16 (9.6%)	16 (9.6%)	7 (4.2%)
Ran out of pills	35 (21.0%)	29 (17.4%)	3 (1.8%)	3 (1.8%)
Wanted to avoid medication adverse effects	29 (17.4%)	15 (9.0%)	8 (4.8%)	6 (3.6%)
Reduced motivation because TB symptoms improved	19 (11.4%)	7 (4.2%)	5 (3.0%)	7 (4.2%)
Had too many pills to take for different conditions	17 (10.2%)	9 (5.4%)	4 (2.4%)	4 (2.4%)
Did not want others to notice me taking medication	16 (9.6%)	12 (7.2%)	2 (1.2%)	2 (1.2%)

^a^Participants could report more than 1 reason for missing doses.

^b^Represents the proportion of study participants reporting a given problem over the total number in the cohort who reported having ever missed medication doses: eg, 67 of 167 participants reported traveling or being away from home as a reason for missing doses.

### Association Between Medication Nonadherence and Tuberculosis Treatment Outcomes

Using Nikshay data, nonadherence had a statistically significant association with loss to follow up and an association with death that did not quite achieve statistical significance (Table 4). Nonadherent participants had 4.0 (95% confidence interval, 2.1–7.6) higher odds of unfavorable treatment outcomes (*P* < .0001). Analyses using suboptimal adherence as the outcome, and using data from treatment cards alone, yielded similar findings (Supplementary Appendix, Tables S3–S5).

**Table 4.** Association Between Medication Nonadherence and TB Treatment Outcomes Reported in India’s National TB Elimination Program Nikshay Electronic Record System for the Cohort (N = 565)^a^

	Descriptive Statistics		Univariable Findings	
Treatment Outcome	Proportion of Sample in Given Category^b^, n (%)	Proportion With Medication Nonadherence^c^, n (%)	Odds Ratio (95% Confidence Interval)	*P* Value
Treatment success (cure or treatment completion)	513 (90.8)	48 (9.4)	Ref	
Died	18 (3.2)	4 (22.2)	2.8 (0.9–8.7)	.08
Lost to follow up	34 (6.0)	11 (32.4)	4.6 (2.1–10.1)	.0001*

Abbreviations: Ref, reference group; TB, tuberculosis.

^a^Sample excludes 85 (13.1%) study participants, including 77 for whom the study closed before they finished treatment and outcomes were also not reported in Nikshay, 5 who underwent a change in treatment regimen, and 3 who experienced treatment failure.

^b^Represents the number of participants in a category divided by the overall cohort sample with available treatment outcomes of 565: eg, 513 of 565 participants experienced treatment success.

^c^Represents the number of participants with nonadherence in a given category: eg, 48 of 513 participants with treatment success were nonadherent.

*Indicates a statistically significant finding at the 5% level.

## DISCUSSION

In this cohort study, we used survey questions and urine metabolite testing, a rigorous marker of isoniazid ingestion, to identify factors associated with nonadherence to drug-susceptible TB treatment in India. We found that medication nonadherence is a complex problem that involves structural, psychosocial, and clinical barriers that may be amenable to intervention.

As found with other TB care cascade gaps, structural barriers—particularly distance from the clinic [33, 34]—may be a central challenge leading to nonadherence. Nonadherence increased considerably for participants who spent 30 minutes or more collecting medication refills—a factor that was collinear with other structural barriers (ie, transportation mode and money spent collecting medications). These structural barriers triangulate with the survey finding that more than one fifth of participants reported running out of pills as a reason for missing doses. Similar to findings in the HIV literature, many patients attributed pill shortages to transportation difficulties or other challenges collecting medications [35].

People with HIV disproportionately experienced these structural barriers, likely related to the single window policy in which PWH now collect TB medications concurrently with their HIV medications at ART centers instead of DOT centers. Although “one-stop shopping” seems to be a laudable goal, creation of new barriers through longer commutes to ART centers may attenuate the benefits of consolidated pharmacy services. Our findings may explain results of a study in Karnataka, India, in which outcomes were worse for PWH receiving TB treatment at ART centers via the single window system, compared with PWH receiving treatment at DOT centers [26].

Interventions addressing structural barriers may include providing travel vouchers to help patients reach clinics or delivering medications to patients. For example, recent studies have evaluated the benefits of HIV adherence clubs, in which PWH meet locally to discuss their care and collect ART, facilitating easy access to medications and peer support [36]. Such interventions merit evaluation in Indian TB patients.

Laborers who receive daily wages had higher nonadherence. This finding remained significant after adjusting for income, which suggests that this risk may be related to wage losses when people leave work to collect medications and other job constraints, rather than poverty in general. Home medication delivery may benefit patients with such jobs. Many patients reported traveling away from home as a reason for missing doses, which may also reflect job-related constraints, a barrier also reported in the HIV literature [37]. Future research should examine strategies to support medication access and adherence during travel.

Psychosocial factors also emerged as important barriers to adherence. Alcohol use was significantly associated with nonadherence, consistent with findings from previous Indian studies [38–40]. Although we did not formally measure depression, more than one fifth of patients who reported missing doses described feeling depressed as a reason. In a recent systematic review, researchers found that depression is associated with increased loss to follow up and death—but not medication nonadherence—during TB treatment; however, the studies that were included had suboptimal measures of adherence [41]. Future studies using rigorous measures of both depression and adherence, such as the urine testing we used here, may clarify whether nonadherence mediates the association between depression and treatment outcomes. Some patients reported missing doses because they did not want others to notice them taking medications, which highlights stigma as another barrier [42, 43].

Clinical factors may also contribute to nonadherence. Smear-positive pulmonary TB patients had higher adjusted odds of nonadherence. These patients have poorer outcomes on average compared with smear-negative and extrapulmonary TB patients in the NTEP [44]. Our finding suggest that poorer treatment outcomes in smear-positive patients could be mediated not only by greater disease severity but also by higher nonadherence. A desire to avoid medication adverse effects was also reported as a reason for missing doses, consistent with previous studies in India [45]. Finally, nonadherence was higher in the late continuation phase of therapy, a finding that was consistent with some patients describing symptom improvement as a reason for missing doses. Enhanced counseling may help to address patient concerns about adverse effects while also increasing motivation to adhere later in the treatment, when symptoms have improved.

Many patients said they simply “forgot” to take medications. Although forgetfulness is often perceived as a cognitive problem, it is also shaped by other barriers. Depression and alcohol use can reduce cognitive function, increasing the chance that patients will forget to take pills, whereas structural barriers to collecting medications may result in patients prioritizing other tasks. One rationale for using DATs is that reminders provided by these technologies might reduce forgetfulness; however, these reminders do not address the contextual barriers that contribute to forgetting medication doses.

Despite only conducting a single urine isoniazid test for each patient, a negative test result was strongly associated with unfavorable treatment outcomes. This finding highlights possible benefits of urine testing not only for research, but also for identifying patients at risk for poor outcomes in routine care. Conducting multiple urine tests throughout therapy might facilitate better prediction of treatment outcomes. However, further research is needed to (1) understand whether urine testing might be acceptable to patients in routine care, (2) evaluate whether tests conducted during prescheduled clinic visits are as accurate as tests conducted during unannounced home visits [18], and (3) ensure that testing does not have the unintended consequence of leading healthcare providers to stigmatize nonadherent patients.

A study limitation is that our cohort may not be representative of the overall TB patient population at these clinics, because 53 of 832 (6%) patients did not consent for the home visit and 31 of 832 (4%) could not enroll because family members collected their medications. We were also unable to complete home visits for 98 of 748 (13%) enrolled participants. We suspect these participants may have been more likely to be nonadherent and to have suffered unfavorable treatment outcomes. As such, adherence and treatment outcomes may be higher in our study cohort, because participants who were successfully enrolled and available for a home visit may have been more motivated to adhere and complete treatment. In addition, because many participants in our cohort had their home visit relatively late in treatment (ie, the continuation phase), the predictive value of the urine test may have been reduced, because these participants were far along the pathway to their treatment outcome.

Our understanding of the influence of HIV infection on TB medication adherence was also limited, because HIV status also served as a proxy for differences in geographic sites and care delivery approaches. Although inclusion of PWH and HIV-negative patients facilitated unique insights, such as the importance of time spent collecting medications, combining these populations may have masked meaningful associations with factors that could be more relevant to one of these groups.

A strength of our study is that we present analyses using nonadherence and suboptimal adherence as outcomes, because these outcomes represent 2 practical ways the urine test could be interpreted in clinical practice. Although the general consistency of findings across these preplanned analyses is reassuring, conducting multiple analyses with several predictors and similar outcome measures may increase risk of type I error—that is, that we incorrectly identified significant associations. In addition, we had a modest number of outcomes (ie, patients with negative urine tests), which may have limited statistical power to identify factors associated with nonadherence. Future studies involving a larger sample, with multiple urine tests on each patient, might identify additional factors associated with nonadherence.

## CONCLUSIONS

In this study of drug-susceptible TB patients in India, we found that nonadherence is a complex problem shaped by structural, psychosocial, and clinical barriers. Structural barriers—in particular, transportation challenges and time and money spent collecting medications—had the strongest association with nonadherence, particularly for PWH. Psychosocial barriers such as alcohol use, depression, and stigma also emerged as major problems contributing to nonadherence.

Our findings highlight a need to facilitate easier access to medication refills and to develop counseling interventions that can address depression, substance use disorders, and stigma. Drug metabolite testing is a useful approach for measuring adherence in TB patients taking SAT- or DAT-monitored therapy, particularly because nonadherence may predict subsequent outcomes in the TB care cascade [46], including unfavorable treatment outcomes and disease recurrence. Future research should focus on assessing whether urine testing can be used to improve adherence in routine care and on evaluating interventions addressing the diverse barriers contributing to TB medication nonadherence in high-burden countries.

## Supplementary Data

Supplementary materials are available at Open Forum Infectious Diseases online. Consisting of data provided by the authors to benefit the reader, the posted materials are not copyedited and are the sole responsibility of the authors, so questions or comments should be addressed to the corresponding author.

ofab190_suppl_Supplementary_Appendix
