# Supplementary material for: Understanding Nonadherence to Tuberculosis Medications in India Using Urine Drug Metabolite Testing: A Cohort Study
Source: Open Forum Infect Dis. 2021 May 5;8(6):ofab190. doi: 10.1093/ofid/ofab190 (PMC8262681; doi:10.1093/ofid/ofab190)
Supplement: ofab190_suppl_Supplementary_Appendix [file ofab190_suppl_supplementary_appendix.docx]

**Supplementary Appendix**

**Supplement to:**

*Understanding non-adherence to tuberculosis medications in India using urine drug metabolite testing: a cohort study*

Ramnath Subbaraman**,** Beena E. Thomas, J. Vignesh Kumar, Kannan Thiruvengadam, Amit Khandewale, S. Kokila, Maya Lubeck-Schricker, M. Ranjith Kumar, Gunjan Rahul Gaurkhede, Apurva Shashikant Walgude, J. Hephzibah Mercy, Jagannath Dattatraya Kumbhar, Misha Eliasziw, Kenneth H. Mayer, Jessica E. Haberer

**Correspondence:** Beena E. Thomas, Department of Social and Behavioural Research, ICMR- National Institute for Research in Tuberculosis, No. 1, Mayor Sathiyamoorthy Road, Chetpet, Chennai – 600 031, India ([beenaelli09@gmail.com](mailto:beenaelli09@gmail.com)). Phone: +91-44-2836-9525

**Methods**

*Rationale for variables included in the regression analyses*

For our multivariable logistic regression models evaluating factors associated with non-adherence and suboptimal adherence, we included variables that represent key demographic and clinical factors that are routinely adjusted for in analyses of TB outcomes and variables that have the potential to influence adherence behavior. Gender and age have been associated with differences in various TB outcomes, including care-seeking, pretreatment loss to follow-up, and mortality [1–3]. Income and occupation have the potential to influence patients’ ability to engage in TB care. For example, patients may not have enough money or may be in jobs that allow limited flexibility to return to the clinic to pick up medication refills or to take medications on time [4].

With regard to clinical variables, phase of therapy represents the timing of the adherence measurement. Medication adherence may vary throughout the TB treatment course, particularly as patients’ symptoms improve or pill fatigue sets in, potentially reducing motivation to adhere to therapy [5]. In India’s NTEP and globally, previously treated patients (as compared to new patients), smear-positive patients (as compared to extrapulmonary or smear-negative patients), and PLHIV (as compared to HIV-negative patients) have relatively poorer TB treatment outcomes [6]. As such, we felt these variables were important to include in our analysis.

Mode of transportation to the clinic, money spent to collect medication refills, and time spent to collect medication refills were variables representing structural challenges that could prevent patients from picking up their medications, thereby affecting their adherence. Tobacco and alcohol are substances that have the potential to affect patient behavior, for example by causing patients to be inebriated, resulting in missed doses. Both have been associated with relatively poorer TB treatment outcomes in prior literature [7,8].

*Addressing multicollinearity in the regression analyses*

When conducting regression diagnostics, HIV status was observed to have a high degree of multicollinearity (variance inflation factor >5) with mode of transport, money spent collecting medication refills, and time spent collecting medication refills. By removing mode of transport and money spent collecting medication refills from the model, we were able to reduce multicollinearity and retain HIV status and time spent collecting medication refills, which is the structural barrier with the strongest association with non-adherence. Similarly, given high multicollinearity between alcohol and tobacco use, we removed tobacco use from our model, because alcohol use seemed more likely to contribute to behavioral changes (e.g., inebriation, inattention, etc.) that could contribute to non-adherence.

*Analysis of the association between non-adherence and treatment outcomes – description of the two data sources*

Treatment card outcomes were collected directly from the paper-based treatment cards maintained by TB staff at DOT centers. These are standardized cards that include basic demographic information about the patients, information on medication refills collected, and treatment outcomes. Possible treatment outcomes recorded on treatment cards are cure, treatment completion, loss to follow-up (formerly default), death, treatment regimen change, transferred out, and still on treatment (Table S1).

Patient outcomes from treatment cards are then entered into Nikshay, the NTEP’s electronic medical record, at a later date, usually by personnel at a centralized location such as a district or city TB office. Because data from paper treatment cards are sometimes entered into Nikshay weeks or months later, we waited to obtain data from Nikshay until September 2020. Possible treatment outcomes recorded in the Nikshay database are cure, treatment completion, loss to follow up, death, treatment regimen change, treatment failure, null, not evaluated, and closed due to inactivity (Table S1). We interpreted the last three of these outcomes (i.e., null, not evaluated, and closed due to inactivity) as representing missing data on treatment outcomes in Nikshay.

Our research team collected treatment outcomes directly from the paper treatment cards through the end of February 2019, when our field research ended. Because our research team was able to collect this information directly from the paper-based records on which patient information is initially recorded, we believe that the treatment card-based outcomes are highly accurate. However, a limitation of the treatment card data is that we were not able to collect final outcomes from treatment cards for 269 patients, because they were still on treatment when the study ended (Table S1). In addition, 10 patients transferred their care to another district, and our study team was therefore not able to verify outcomes for treatment cards for these patients. Four patients underwent a change in treatment regimen. Treatment regimen change may occur multiple reasons—including medication adverse effects or diagnosis of drug resistance, in addition to treatment failure. As such, given that treatment regimen change does not necessarily indicate an unfavorable outcome, we also excluded these patients from the analysis of data from treatment cards.

Due to this limitation of the treatment card data, we also conducted analyses using data from Nikshay, which recorded patient treatment outcomes beyond the end of our field research. In rare cases when outcomes reported on the treatment cards conflicted with the Nikshay data, we substituted the outcome recorded by Nikshay with the treatment card outcome, because we believe the treatment card data were less likely to be incorrect due to data entry errors. Only 5 (0.8%) patients had treatment outcomes in Nikshay that were discordant with what was reported on patients’ treatment cards (Table S1).

Unfortunately, as of September 2020, treatment outcomes were not entered in Nikshay for 133 (20.5%) patients in our cohort. For these patients, we substituted outcomes collected from paper treatment cards, if available. Notably, 69 (10.6%) patients still had missing outcomes after substituting treatment card outcomes and were excluded from our analysis using Nikshay data. We also excluded patients who underwent treatment regimen changes, without another recorded outcome, because treatment regimen changes do not necessary indicate an unfavorable outcome as noted above. We did not assess the association between non-adherence and treatment failure as a separate outcome, because only three patients experienced treatment failure; however, we did include these patients in the analysis of “unfavorable outcomes” as a composite outcome along with death and loss to follow-up.

**Table S1. Comparison of treatment card and Nikshay data sources**

|  | **Treatment card outcomes** | **Nikshay outcomes** |
| --- | --- | --- |
| **Description of data source** | Standardized paper-based cards maintained by TB staff at DOT centers that include basic demographic information about the patients, information on medication refills collected, and treatment outcomes. | Patient outcomes from treatment cards entered into Nikshay, the NTEP’s electronic medical record, at a later date, usually by personnel at a centralized location such as a district or city TB office. |
| **Possible treatment outcomes recorded in each dataset** | - Cure - Treatment completion - Loss to follow-up - Death - Treatment regimen change - Transferred out - On treatment | - Cure - Treatment completion - Loss to follow-up - Death - Treatment regimen change - Treatment failure - Null - Not evaluated - Closed due to inactivity |
| **Limitations of data source** | Research team was not able to collect final outcomes from treatment cards for patients who were still on treatment when the study ended in February 2019. | Treatment outcomes were missing in Nikshay for 133 (21%) patients in our cohort. If available, we substituted outcomes collected from paper treatment cards for the missing data. Five (0.8%) patients had treatment outcomes in Nikshay that were discordant with what was reported on patients’ treatment cards. In these cases, Nikshay outcomes were substituted with treatment card outcomes. |
| **Approach to handling missing outcomes** | We excluded 283 (43.5%) patients,  including 269 for whom the NIRT study closed before they finished treatment, 10 who had their care transferred out to other districts, and 4 who underwent a change in treatment regimen. | We excluded 85 (13.1%) patients from the final analysis, of whom 77 did not have treatment outcomes available even after substitution of outcomes from treatment cards, 5 underwent a change in treatment regimen, and 3 experienced treatment failure. |

**Results**

*Patient recruitment and data collection*

**
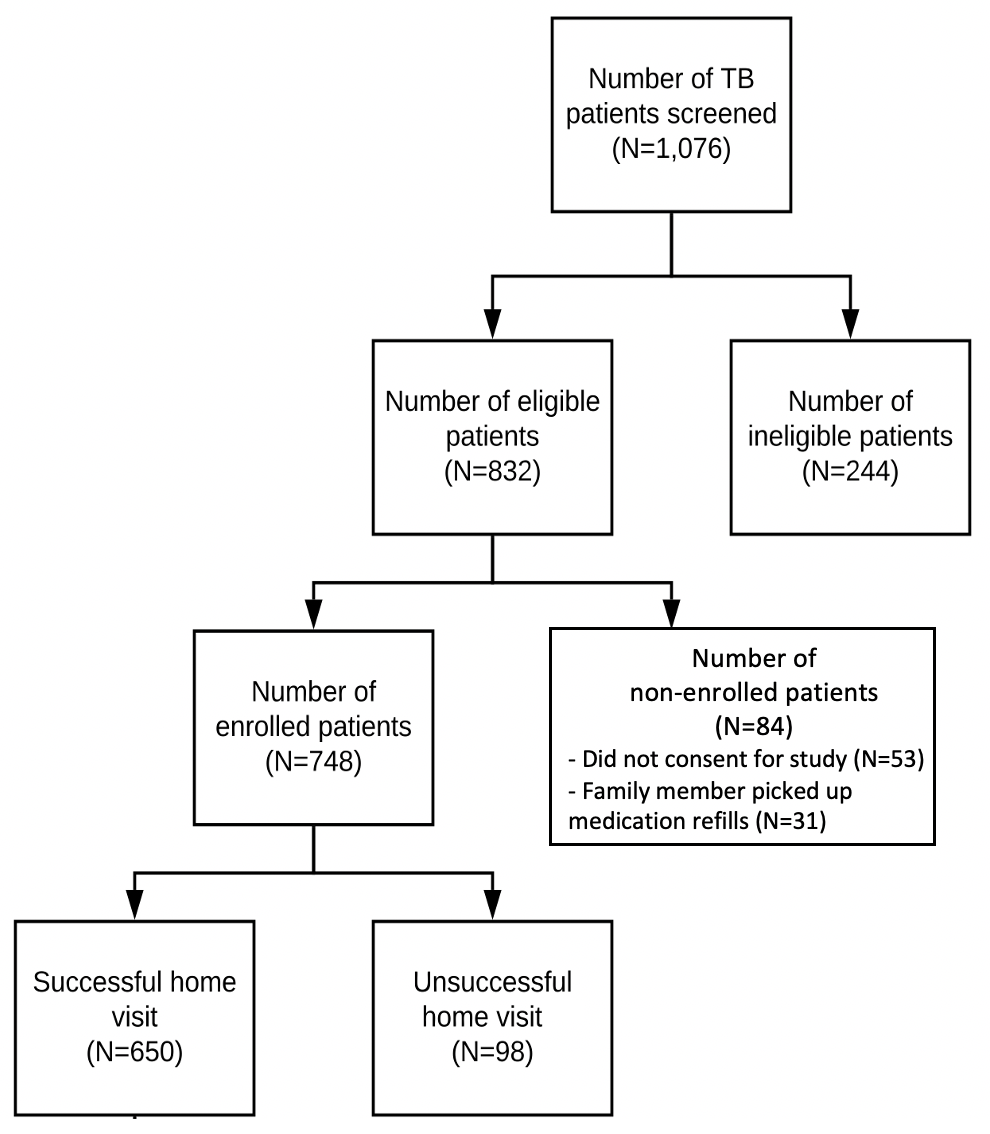
**

**Figure S1: Patients screened, enrolled, and home visits completed in the study cohort.** Patients with unsuccessful home visits were only classified as such after three unsuccessful home visit attempts by the research team.

*Factors associated with non-adherence to TB medications – univariable analysis findings*

In univariable analyses with non-adherence as the outcome, individuals who were 30—44 years old, laborers on daily wages, smear-positive pulmonary TB patients, PLHIV, taking public transportation to collect medication refills, spending 50 or more rupees to collect medication refills, taking 30 or more minutes to collect medication refills, using cigarettes or beedis, and using alcohol were at significantly increased odds of being non-adherent (main manuscript, Table 2).

*Factors associated with suboptimal adherence to TB medications – univariable analysis findings and table with findings from univariable and multivariable analyses*

In univariable analyses with suboptimal medication adherence as the outcome, patients who are laborers on daily wages, take public transportation to collect their medications, spend 50 or more rupees to collect their medication, take 30 or more minutes to collect their medication, and use alcohol are statistically significantly more likely to be sub-optimally adherent (Table S2). Patients with an income >=15,000 rupees are significantly less likely to be sub-optimally adherent (Table S2). Findings of the multivariable analysis are shown in Table S2 below and described in the main text of the manuscript.

Table S2. Factors associated with suboptimal adherence to TB medications (N=650)

| **Covariates** | **Descriptive Statistics** | **Univariable findings** | | **Multivariable findings** | |
| --- | --- | --- | --- | --- | --- |
|  | **Proportion of suboptimal adherence**^a^  **n (%)** | **Odds ratio (confidence interval)** | **p-value** | **Odds ratio (confidence interval)** | **p-value** |
| **DEMOGRAPHIC FACTORS** |  |  |  |  |  |
| **Gender** |  |  |  |  |  |
| Female | 49 (18.1) | Ref |  | Ref |  |
| Male | 67 (17.7) | 1.0 (0.6—1.5) | 0.90 | 0.8 (0.5—1.4) | 0.46 |
| **Age** |  |  |  |  |  |
| 18-29 | 33 (14.6) | Ref |  | Ref |  |
| 30-44 | 53 (21.1) | 1.6 (0.97—2.5) | 0.07 | 0.9 (0.5—1.6) | 0.75 |
| ≥45 | 30 (17.3) | 1.2 (0.7—2.1) | 0.46 | 0.7 (0.4—1.4) | 0.35 |
| **Monthly income** |  |  |  |  |  |
| <7500 | 53 (21.7) | Ref |  | Ref |  |
| 7500—14,999 | 46 (17.5) | 0.8 (0.5—1.2) | 0.23 | 1.1 (0.7—1.8) | 0.73 |
| >=15,000 | 17 (11.9) | 0.5 (0.3—0.9) | 0.02* | 0.7 (0.4—1.4) | 0.31 |
| **Occupation** |  |  |  |  |  |
| Self-employed | 18 (13.1) | Ref |  | Ref |  |
| Employed in government or private sector | 19 (14.6) | 1.1 (0.6—2.3) | 0.73 | 1.3 (0.6—2.7) | 0.48 |
| Laborer on daily wages | 23 (27.4) | 2.5 (1.3—5.0) | 0.009* | 2.7 (1.3—5.6) | 0.01* |
| Housewife, student, or unemployed | 56 (18.7) | 1.5 (0.9—2.7) | 0.15 | 1.7 (0.8—3.3) | 0.14 |
| **CLINICAL FACTORS** |  |  |  |  |  |
| **Phase of therapy** |  |  |  |  |  |
| Intensive phase | 32 (18.0) | Ref |  | Ref |  |
| Early continuation phase | 32 (12.9) | 0.7 (0.4—1.1) | 0.15 | 0.9 (0.5—1.6) | 0.71 |
| Late continuation phase | 52 (23.3) | 1.4 (0.8—2.3) | 0.19 | 1.7 (0.99—2.9) | 0.06 |
| **Category of TB** |  |  |  |  |  |
| New | 87 (17.3) | Ref |  | Ref |  |
| Previously treated | 29 (19.9) | 1.2 (0.7—1.9) | 0.47 | 1.1 (0.7—1.9) | 0.60 |
| **Type of TB** |  |  |  |  |  |
| Extrapulmonary | 32 (15.8) | Ref |  | Ref |  |
| Smear-negative pulmonary | 17 (22.1) | 1.5 (0.8—2.9) | 0.22 | 1.3 (0.6—2.5) | 0.52 |
| Smear-positive pulmonary | 67 (18.1) | 1.2 (0.7—1.9) | 0.50 | 1.2 (0.7—1.9) | 0.52 |
| **Living with HIV** |  |  |  |  |  |
| No | 43 (12.4) | Ref |  | Ref |  |
| Yes | 73 (24.1) | 2.2 (1.5—3.4) | 0.0001* | 1.5 (0.7—3.3) | 0.31 |
| **STRUCTURAL FACTORS** |  |  |  |  |  |
| **Mode of transport to treatment site** |  |  |  |  |  |
| Walking or bicycle | 26 (11.1) | Ref |  |  |  |
| Motorcycle or car | 6 (13.6) | 1.3 (0.5—3.3) | 0.63 |  |  |
| Autorickshaw or taxi | 22 (16.8) | 1.6 (0.9—3.0) | 0.13 |  |  |
| Public transportation | 62 (25.7) | 2.8 (1.7—4.6) | <0.0001* |  |  |
| **Money spent to collect medications** |  |  |  |  |  |
| 0—24 | 30 (12.9) | Ref |  |  |  |
| 25—49 | 15 (13.5) | 1.1 (0.5—2.1) | 0.87 |  |  |
| 50—75 | 24 (21.6) | 1.9 (1.0—3.4) | 0.04* |  |  |
| >75 | 47 (24.1) | 2.1 (1.3—3.6) | 0.003* |  |  |
| **Time spent to collect medication** |  |  |  |  |  |
| <30 minutes | 4 (3.6) | Ref |  | Ref |  |
| 30 to 59 minutes | 32 (16.8) | 5.3 (1.8—15.5) | 0.002* | 5.7 (1.9—16.8) | 0.002* |
| >=60 minutes | 80 (22.9) | 7.9 (2.8—22.1) | <0.001* | 6.3 (1.9—20.9) | 0.003* |
| **PSYCHOSOCIAL FACTORS** |  |  |  |  |  |
| **Current tobacco use** |  |  |  |  |  |
| No | 93 (17.2) | Ref |  |  |  |
| Smokeless tobacco use only | 7 (13.7) | 0.8 (0.3—1.8) | 0.53 |  |  |
| Cigarettes or beedi use | 16 (27.1) | 1.8 (1.0—3.3) | 0.06 |  |  |
| **Probable alcohol use** |  |  |  |  |  |
| No alcohol use | 96 (16.2) | Ref |  | Ref |  |
| Any alcohol use | 20 (33.9) | 2.6 (1.5—4.7) | 0.001* | 2.3 (1.2—4.5) | 0.01* |

INR=Indian rupees.

^a^Proportion represents the number of participants with suboptimal adherence in a given category—e.g., 49/271 females were suboptimally-adherent.

*Patient-reported reasons for non-adherence: details on reasons patients ran out of pills and medication adverse effects experienced*

Among 35 patients who reported non-adherence because they ran out of pills, 12 (34.3%) reported that this was related to difficulties in picking up medication refills (e.g., transportation challenges, illness), 8 (22.9%) forgot to pick up their medication refills, 6 (17.1%) went to pick up a refill but found that the healthcare provider was absent or the clinic was out of stock of medications, 5 (14.3%) went to pick up a refill but found the clinic was closed due to a holiday, 2 (5.7%) could not pick up medications due to travel out of town, and 2 (5.7%) reported other personal barriers to picking up medication refills. Among 29 patients who reported non-adherence due to fear of medication side effects, 10 (34.5%) had experienced nausea, 5 (17.2%) had experienced fatigue, 3 (10.3%) had experienced rash, 7 (24.1%) experienced other side effects (e.g., jaundice, giddiness, fever), and 4 (13.8%) reported a general concern that TB medications are “harmful.”

*Association between non-adherence to medications and treatment card-recorded TB treatment outcomes*

Using the treatment outcomes collected on treatment cards, 367 (56.4%) patients in the cohort had treatment outcomes available. Patients who were lost to follow-up had statistically significantly higher odds of being non-adherent (Table S3). The outcome of death was also associated with increased odds of non-adherence, although this did not achieve statistical significance.

We also evaluated the association between suboptimal adherence and treatment outcomes using outcomes reported by Nikshay (Table S4) and on treatment cards (Table S5). Suboptimal adherence was statistically significantly associated with both patient loss to follow-up and death in both of these analyses.

Table S3. Association between treatment outcomes recorded on treatment cards and medication non-adherence (N=367)^a^

| **Treatment outcomes** | **Descriptive statistics** | | **Univariable findings** | |
| --- | --- | --- | --- | --- |
|  | **Proportion of overall sample in given category**^b^  **n (%)** | **Proportion of medication non-adherence**^c^  **(negative urine INH test result)**  **n (%)** | **Odds ratio**  **(95% confidence interval)** | **p-value** |
| Treatment success  (cure or treatment completed) | 332 (90.5) | 35 (10.5) | Ref |  |
| Died | 15 (4.1) | 4 (26.7) | 3.1 (0.9—10.2) | 0.07 |
| Lost to follow-up | 20 (5.4) | 8 (40.0) | 5.7 (2.2—14.8) | 0.0004* |

^a^Sample excludes 283 (43.5%) patients, including 269 for whom the NIRT study closed before they finished treatment, 10 who had their care transferred out to other districts, and 4 who underwent a change in treatment regimen.

^b^Proportion represents the number of participants in a category divided by the overall cohort sample—e.g., 332/367 participants experienced treatment success.

^c^Proportion represents the number of participants with non-adherence in a given category—e.g., 35/332 participants with treatment success were non-adherent.

Table S4. Association between suboptimal adherence to tuberculosis (TB) medications and treatment outcomes reported in India’s National TB Elimination Program Nikshay system for the cohort (N=565)^a^

| **Treatment outcome** | **Descriptive statistics** | | **Univariable findings** | |
| --- | --- | --- | --- | --- |
|  | **Proportion of overall sample in given category**^b^  **n (%)** | **Proportion with suboptimal adherence**^c^  **n (%)** | **Odds ratio**  **(95% confidence interval)** | **p-value** |
| Treatment success  (cure or treatment completion) | 513 (90.8) | 72 (14.0) | Ref |  |
| Died | 18 (3.2) | 7 (38.9) | 3.9 (1.5—10.4) | 0.0065* |
| Lost to follow-up | 34 (6.0) | 13 (38.2) | 3.8 (1.8—7.9) | 0.0004* |

^a^Sample excludes 85 (13.1%) patients, including 77 for whom the NIRT study closed before they finished treatment and outcomes were also not reported in Nikshay, 5 who underwent a change in treatment regimen, and 3 who experienced treatment failure.

^b^Represents the number of participants in a category divided by the overall cohort sample with available treatment outcomes of 565—e.g., 513/565 participants experienced treatment success.

^c^Represents the number of participants with suboptimal adherence in a given category—e.g., 72/513 participants with treatment success were non-adherent.

Table S5. Association between treatment outcomes recorded on treatment cards and suboptimal medication (N=367)^a^

| **Treatment outcomes** | **Descriptive statistics** | | **Univariable findings** | |
| --- | --- | --- | --- | --- |
|  | **Proportion of overall sample in given category**^b^  **n (%)** | **Proportion with suboptimal adherence**^c^  **n (%)** | **Odds ratio**  **(95% confidence interval)** | **p-value** |
| Treatment success  (cure or treatment completed) | 332 (90.5) | 53 (16.0) | Ref |  |
| Died | 15 (4.1) | 7 (46.7) | 4.6 (1.6—13.2) | 0.005* |
| Lost to follow-up | 20 (5.5) | 10 (50.0) | 5.3 (2.1—13.3) | 0.0004* |

^a^Sample excludes 283 (43.5%) patients, including 269 for whom the NIRT study closed before they finished treatment, 10 who had their care transferred out to other districts, and 4 who underwent a change in treatment regimen.

^b^Proportion represents the number of participants in a category divided by the overall cohort sample—e.g., 332/367 participants experienced treatment success.

^c^Proportion represents the number of participants with non-adherence in a given category—e.g., 35/332 participants with treatment success were non-adherent.

**References**

1. Horton KC, White RG, Houben RMGJ. Systematic neglect of men as a key population in tuberculosis. Tuberculosis (Edinb) **2018**; 113:249–253.

2. Chikovore J, Pai M, Horton KC, et al. Missing men with tuberculosis: the need to address structural influences and implement targeted and multidimensional interventions. BMJ Glob Health **2020**; 5:e002255.

3. Thomas BE, Subbaraman R, Sellappan S, et al. Pretreatment loss to follow-up of tuberculosis patients in Chennai, India: a cohort study with implications for health systems strengthening. BMC Infect Dis **2018**; 18:142.

4. Bhargava A, Bhargava M, Juneja A. Social determinants of tuberculosis: context, framework, and the way forward to ending TB in India. Expert Rev Respir Med [Internet] **2020**. Epub ahead of print 2020 Oct 20. doi: [10.1080/17476348.2021.1832469](https://doi.org/10.1080/17476348.2021.1832469).

5. Paunikar AP, Khadilkar HA, Doibale MK, Lamb AR. Survival Analysis of Treatment Defaulters among Tuberculosis Patients in Government Medical College and Hospital, Aurangabad. Indian J Community Med **2019**; 44:44–47.

6. India TB Report 2020. Nirman Bhawan, New Delhi: Central TB Division, Ministry of Health and Family Welfare, 2020. Available at: https://tbcindia.gov.in/WriteReadData/l892s/India%20TB%20Report%202020.pdf. Accessed 17 March 2021.

7. Thomas BE, Thiruvengadam K, S R, et al. Smoking, alcohol use disorder and tuberculosis treatment outcomes: A dual co-morbidity burden that cannot be ignored. PLoS One **2019**; 14:e0220507.

8. Ma Y, Che N-Y, Liu Y-H, et al. The joint impact of smoking plus alcohol drinking on treatment of pulmonary tuberculosis. Eur J Clin Microbiol Infect Dis **2019**; 38:651–657.
